# Retention in HIV care and associated factors among youths aged 15–24 years in rural southwestern Uganda

**DOI:** 10.1186/s12889-021-11547-5

**Published:** 2021-07-31

**Authors:** Moses Muwanguzi, Henry Mark Lugobe, Elastus Ssemwanga, Allan Phillip Lule, Elizabeth Atwiine, Vincent Kirabira, Ann K. Stella, Scholastic Ashaba, Godfrey Zari Rukundo

**Affiliations:** 1 Mbarara University of Science and Technology, Faculty of Medicine, Mbarara, Uganda; 2 Department of Obstetrics and Gynecology, Mbarara University of Science and Technology, Mbarara, Uganda; 3 Department of Psychiatry, Mbarara University of Science and Technology, Mbarara, Uganda

**Keywords:** HIV/AIDS, HIV stigma, Retention in HIV care, Youths, ART

## Abstract

**Background:**

Retention in HIV care contributes to antiretroviral therapy adherence, which is a key factor for improved treatment outcomes and prevention of drug resistance. However, HIV treatment among the youths is characterized by loss to follow up, poor adherence to ART, risk of treatment failure and high mortality rates compared to young children and adults. There is limited information about factors associated with retention of youths in HIV care in rural settings in Uganda. We aimed to determine retention in HIV care and associated factors among youths aged 15–24 years in rural southwestern Uganda.

**Methods:**

A cross-sectional study was conducted among youths aged 15–24 years who were receiving care at the HIV clinic at Kabuyanda HC IV who had been in care for at least 1 year before the study. We used an interviewer-administered questionnaire to collect socio-demographic information. Participant chart abstraction was used to collect information on HIV clinic attendance. We collected information on HIV related stigma using the 40-item Berger Stigma Scale. Chi-square test and multivariable logistic regression analysis were used to determine the factors associated with retention in HIV care with a significance level of < 0.05. Retention in HIV care was, defined as having sought care at least once per quarter in the 12 months prior to the study.

**Results:**

We enrolled 102 participants with a mean age of 20.95 (SD ± 3.07) years. Two thirds (65.7%) of the youths had been retained in HIV care in the previous 12 months. In adjusted analyses, being male, married and had perinatally acquired HIV were independently associated with retention in HIV care. The association between HIV related stigma and retention in HIV care was not statistically significant.

**Conclusion:**

Retaining adolescents and young adults in HIV care in rural southwestern Uganda is still much lower than the WHO target of 90%. Being male, having perinatally acquired HIV and married or in a relationship are associated with retention in HIV care. Interventions targeting adolescents and young adults living with HIV are necessary to improve retention in HIV care to the WHO target of 90%.

**Supplementary Information:**

The online version contains supplementary material available at 10.1186/s12889-021-11547-5.

## Background

Retention in HIV care is essential and provides opportunities to monitor response to therapy, prevent associated complications, and deliver ancillary services [1]. Retention in HIV health care services is a critical precursor to antiretroviral therapy (ART) adherence and viral suppression [2]. To prevent considerable HIV related morbidity and mortality, all HIV-positive persons must be put on ART and should remain in care to achieve virologic suppression [3]. According to the UNAIDS 90–90-90 strategy, 90% of those enrolled into HIV care should be retained. However, this has not yet been achieved in many settings and sub-populations including adolescents and young adults [4, 5]. Few HIV positive youths below 25 years are retained in care after initiation of ART [6–8]. The poor retention in HIV care could be due to social, structural or health-related factors such as stigma and discrimination, distance and transport to the health facility, poverty and unemployment, work/child care responsibilities, and social relations as major determinants of retention in HIV care [9]. A recent study by Izudi and colleagues [10] in Uganda found that approximately 70% of adolescents were non-retained in care at Katooke Health Center, mid-western Uganda. Poor retention in care has been associated with the duration on ART and adolescent age, with older adolescents (15–19 years) having poorer retention in HIV care compared to younger adolescents aged 10–14 years [11]. Yet, suboptimal retention in HIV care is associated with poor ART adherence and suboptimal virologic non-suppression which worsen health outcomes [12, 13]. This in turn leads to increased HIV associated morbidity and mortality, and poor quality of life [14].

Previous studies in low and middle-income countries have observed that factors influencing retention in care are similar to those that influence adherence to ART [15, 16]. Factors associated with retention in HIV care have been categorized into individual, political, socio-economic, stigma and discrimination, sociodemographic and health system factors [17]. In Zambia, a mixed study to examine barriers to retention in HIV care of HIV positive adolescents identified multiple factors including stigma and discrimination, poverty, disrespectful treatment from clinicians, their adolescent-specific responsibilities (e.g. school), and cultural beliefs and traditions about illness [18]. However, there is a paucity of information about factors associated with retention in HIV care services among youths aged 15–24 years residing in rural settings of low income countries. Therefore, we aimed to determine retention in HIV care and associated factors among HIV positive youths aged 15–24 years enrolled for HIV care in rural southwestern Uganda.

## Methods

### Study design & site

We conducted a cross-sectional study at an HIV clinic at Kabuyanda Health Centre IV in Isingiro district from July 2020 to August 2020. The health facility is located 108 Km south of Mbarara city. Kabuyanda HC IV is the only public health facility in the area that offers HIV care services. The adult HIV clinic operates 2 days a week (Wednesday and Thursday) for Antiretroviral Therapy (ART) refill appointments but the adolescent HIV clinic operates every last Thursday of the month. At Kabuyanda HC IV, clients are given review visits depending on their clinical status, where stable and virally suppressed individuals are reviewed every 90 days. Clients who are clinically unstable and those with unsuppressed viral loads have their review visits individualized in a range of 30–60 days. During the nationwide lockdown in Uganda (from March 2020 to July 2020), the health workers and clinic peer educators at the clinic took responsibility of delivering drugs to the clients’ homes. The clinic serves about 1200 HIV clients of whom 112 are youths aged 15–24 years. Isingiro district was selected for this study due to its young population: 57.2% aged 0–17 years and 17.3% aged 15–24 years [19]. The study area is a rural community with households earning their livelihood through subsistence farming and cattle keeping.

### Study participants

The study participants were youth aged 15–24 years living with HIV who were accessing care at Kabuyanda Health Centre IV. We included youths who had been in care at the facility for at least 1 year. The youth who were less than 1 year in care at the facility at the time of the study were excluded.

### Sample size

For this study, we screened all youth attending the HIV clinic at Kabuyanda HC IV for eligibility. Therefore a sample size for this study was not calculated a priori.

### Sampling and data collection

A list of potential participants was generated by the data clerks and the HIV counsellor at the facility contacted the participants by phone calls and referred them to the research team at the facility who assessed their eligibility to participate in the study. Those who met the inclusion criteria, study aims were explained to them in their local language and were given a chance to ask questions for clarification. All eligible participants gave written informed consent to participate in this study. Participants below 18 years assented and their caregivers provided written informed consent. A consecutive sample of youths aged 15–24 years who consented to participate in the study were recruited.

### Study variables

#### Retention HIV in care

Our primary outcome variable was retention in HIV care. According to the Uganda ministry of health, retention HIV in care is defined as the number of HIV positive persons with at least 1 HIV clinic visit within 90 days [10]. Therefore, in this study to determine retention in HIV care within a 1-year period, we defined retention as having attended at least 4 visits in 12 months prior to this study with at least 1 visit each quarter. The 12 months were divided into four equal quarters with each quarter consisting 90 days. The number of visits in each quarter was recorded. We reviewed participants’ medical charts of 12 months before the study and each medical chart was assigned a code to ensure confidentiality. The number of clinic visits was abstracted from each participant’s medical chart.

#### Socio-demographic and disease characteristics

A well-designed interviewer-administered questionnaire was created (S1 Text) to explore factors that influence retention in HIV Care. The tool was designed to capture the socio-demographic information including; age, gender, occupation, marital status, duration on treatment, ART regimen, whether perinatally acquired HIV (this was a verbal report of whether one was perinatally acquired HIV or not) and HIV disclosure status.

#### HIV Berger Stigma Scale

HIV-related stigma for each participant was measured using the Berger HIV Stigma Scale [20], which is a validated and standardized measure of stigma experienced by people living with HIV. It contains 40-items scored on a 4-point Likert-type scale (strongly disagree, disagree, agree, strongly agree) with total stigma scores ranging from 40 to 160. The scale measures 4 stigma subscales; (1)personalized stigma (assessed by 18 items) measuring consequences of people knowing ones HIV status including rejection by others, loss of close friends, (2) disclosure concerns (assessed by 10 items) which measure the likelihood that one will tell others about their HIV diagnosis, (3) negative self-image (assessed by 13 items) assessing individual’s feelings about themselves, (4) concern with public attitudes (assessed by 20 items) which measures participants’ public’s perceptions of attitudes towards persons living with HIV [20]. Higher scores indicate a greater level of agreement with each item, and the severity of stigma.

#### Data collection

The Interviewer administered questionnaire and the Berger stigma scale were administered in the same interview with each participant. The duration of the interview was approximately 35–45 min. Interviews were conducted in a doctor’s room at the health facility to ensure privacy and confidentiality of patients’ information. Participants who required post-interview counselling were referred to the facility counsellor after the interview.

### Data management

At the end of every interview, the questionnaires were reviewed for completeness. The research team ensured that patient charts were de-identified and reviewed only once. Filled questionnaires were kept in a lockable cupboard to ensure data safety. Data entry forms were prepared in Microsoft excel 2013 where data were entered in duplicate to avoid errors. Entered data was saved and stored on a password-protected computer that was only accessed by the research team members. A copy of the same data set file was saved on a Flash disk stored by the principal investigator, as a back-up file. After data cleaning, data were exported to Stata version 15 (Statacorp, College Station, TX, USA) for analysis.

### Data analysis

Descriptive statistics for categorical variables were presented in frequency tables while continuous variables were described using means and standard deviations. Retention in HIV care was calculated as the proportion of youths aged 15–24 years who sought care from Kabuyanda HC IV at least once each quarter in the 12 months prior to the study out of the total number of participants. The age of participants was stratified into adolescents (15–19 years) and young adults (20–24 years). The total Berger stigma scale score and sub-scores of individual forms of stigma were obtained by adding Likert scores for individual items in the scale. Due to the lack of a universally accepted cut point of the scores, we adopted the categorization put forward by Charles and colleagues [21] in which the overall stigma scores were categorized into three categories as no/mild, moderate, and severe stigma using the 33rd and 66th percentile cut off values from the distribution of scores. From this, we obtained proportions of youths experiencing different levels of stigma. Considering the possible stigma scores for total stigma and the categories, participants who scored below the 33rd percentile of the stigma scale, were considered having no/mild stigma, those scored between 33rd and 66th percentile had Moderate stigma, and those above whose scores are above the 66th percentile had severe stigma. The proportion of participants with stigma was calculated as the total participants with moderate or severe stigma out of the total number of participants. The proportion of participants with different dimensions of stigma was calculated as the total participants with moderate or severe specific stigma dimension out of the total number of participants.

At bivariate analysis, we analyzed categorical variables using cross-tabulations, crude odds Ratios (cOR) and a chi-square test to assess for the association between the participant characteristics (age, gender, occupation, marital status, geographical location, and disclosure status), HIV related stigma and the likelihood of retention in HIV care. All variables with a *P* value < 0.2, biological plausibility (age, level of education, HIV status disclosure and the four stigma subscales which were; personalized, disclosure concerns, negative self-image, and public attitudes) based on previous literature were considered for the multivariable logistic regression model. We performed a stepwise and backward selection procedure to determine the final parsimonious model of the independent factors associated with our outcome of interest. Confounding and interaction were assessed and the final model checked for goodness of fit using the Hosmer Lemeshow test. The Adjusted Odds Ratios (AOR) with their corresponding 95% confidence interval were presented. A *P* value ≤0.05 was considered statistically significant.

## Results

### Socio-demographics and disease characteristics

The clinic consisted of a total of 112 youths aged 15–24 years in HIV care. Of these, 10 participants had been in HIV care for less than 12 months and were excluded from the study. We therefore enrolled a total of 102 participants in our study. The proportion of youth who were retained in HIV care over a 12 months period was 65.7%. Of the participants who were enrolled into the study, the mean age was 20.95 (SD ± 3.07) ranging from 15 to 24 years. The majority of the participants (77.5%) were female, 74.5% aged 20–24 years, and 69% had attained primary level of education as shown in Table 1.

**Table 1:** Socio-demographic and clinical characteristics of Youths aged 15–24 years receiving ART Care (N-102)

Characteristics	Categories	n (%)	Retention in HIV care		***P***-value
			Yes n (%)	No n (%)	
Age (years)	15–19 years	26 (25.5)	18 (69.2)	8 (30.8)	0.66
	20–24 years	76 (74.5)	49 (64.5)	27 (35.5)	
Sex	Female	79 (77.5)	47 (59.5)	32 (40.5)	0.01
	Male	23 (22.5)	20 (87.0)	3 (13.0)	
Marital status	Married	37 (36.3)	28 (75.7)	9 (24.3)	0.11
	Unmarried	65 (63.7)	39 (60.0)	26 (40.0)	
Level of education	None	17 (16.7)	11 (64.7)	6 (35.3)	0.99
	Primary	71 (69.6)	47 (66.2)	24 (33.8)	
	Secondary	14 (13.7)	9 (64.3)	5 (35.7)	
Geographical location	Kabuyanda SC	49 (48.0)	33 (67.3)	16 (32.7)	0.49
	Kabuyanda TC	15 (14.7)	12 (80.0)	3 (20.0)	
	Kikagati SC	26 (25.5)	15 (57.7)	11 (42.3)	
	Other	12 (11.8)	7 (58.3)	5 (41.7)	
HIV status Disclosure	No	17 (16.7)	9 (52.9)	8 (47.1)	0.23
	Yes	85 (83.3)	58 (68.2)	27 (31.8)	
Perinatally acquired HIV	Yes	19 (18.6)	17 (89.5)	2 (10.5)	0.02
	No	83 (81.4)	50 (60.2)	33 (39.8)	
Total stigma score	Mild/Moderate	42 (41.2)	29 (68.3)	13 (31.7)	0.14
	Severe	60 (58.8)	38(63.3)	22(36.7)	
Personalized stigma	Mild/Moderate	50 (49.1)	30(60.0)	20(40.0)	0.24
	Severe	52 (51)	37(71.2)	15(28.8)	
Disclosure concerns	Moderate	29 (28.4)	20(69.0)	9(31.0)	0.66
	Severe	73 (71.6)	47(64.4)	26(35.6)	
Negative self-image	Moderate	53 (52)	36 (67.9)	17(32.1)	0.62
	Severe	49 (48)	31(63.3)	18(36.7)	
Public attitudes	Moderate	38 (37.3)	24 (63.2)	14 (36.8)	0.68
	Severe	64 (62.7)	43 (67.2)	21(32.8)	

*Legend*: *ART* Anti retroviral therapy, *HIV* Human immunodeficiency virus, *TC* Town Council, *SC* Sub-county

### Level of HIV related stigma

The overall stigma scores ranged from 41 to 154 with a mean score of 112.25 ± 21.77 (95% CI: 107.92, 116.48). The mean scores for the 4 stigma subscales were; personalized (49.03 ± 10.39), disclosure concerns (29.71 ± 5.53), negative self-image (34.60 ± 7.01), and public attitudes (56.60 ± 11.15). According to total stigma scores, 58.8% were severely stigmatized where as 41.2% experienced mild to moderate HIV related stigma. Majority of the participants experienced severe forms of stigma related to disclosure concerns (71.6%) but comparatively lower for negative self-image (48%).

In adjusted analyses, being male (aOR: 5.52, 95% CI 1.28, 23.82, *p* < 0.02), married (aOR: 3.97, 95% CI 1.42, 11.13, *p* < 0.01) and those who perinatally acquired HIV (aOR: 7.23, 95% CI 1.16, 45.07, *p* < 0.03) were independently associated with retention in HIV care as shown in Table 2.

**Table 2:** Bivariate and Multivariate regression analysis of factors associated with retention in HIV care of youths aged 15–24 years (*N* = 102)

Characteristics	Categories	Retention in HIV care		Crude Odds Ratio (95% CI)	***P*** value	Adjusted Odds Ratio (95% CI)	***P*** value
		Yes n (%)	No n (%)				
Age (years)	15–19 years	18 (69.2)	8 (30.8)	ref		ref	
	20–24 years	49 (64.5)	27 (35.5)	0.81 (0.31,2.10)	0.66	1.78 (0.47,6.80)	0.40
Sex	Female	47 (59.5)	32 (40.5)	ref		ref	
	Male	20 (87.0)	3 (13.0)	4.54(1.24,16.55)	0.02	**5.52 (1.28,23.82)**	**0.02**
Marital status	Unmarried	39 (60.0)	26 (40.0)	ref		ref	
	Married	28 (75.7)	9 (24.3)	2.07 (0.84,5.10)	0.11	**3.97 (1.42,11.13)**	**0.01**
Level of Education	None	11 (64.7)	6 (35.3)	ref			
	Primary	47 (66.2)	24 (33.8)	1.07(0.35,3.24)	0.91		
	Secondary	9 (64.3)	5 (35.7)	0.98(0.22,4.30)	0.98		
Perinatally acquired HIV	No	50 (60.2)	33 (39.8)	ref		ref	
	Yes	17 (89.5)	2 (10.5)	5.61(1.22,25.89)	0.03	**7.23 (1.16,45.07)**	**0.03**
Personalized stigma	Mild/Moderate	30(60.0)	20 (40.0)	ref			
	Severe	37(71.2)	15 (28.8)	1.64 (0.72,3.75)	0.24		
HIV status disclosure	No	9 (52.9)	8 (47.1)	ref		ref	
	Yes	58 (68.2)	27 (31.8)	1.91(0.66,5.49)	0.23	2.06 (0.64,6.66)	0.22
Disclosure concerns	Moderate	20(69.0)	9(31.0)	ref			
	Severe	47(64.4)	26(35.6)	0.81(0.32,2.04)	0.66		
Negative self-image	Moderate	36(67.9)	17(32.1)	ref			
	Severe	31(63.3)	18(36.7)	0.81(0.36,1.84)	0.62		
Public attitudes	Moderate	24(63.2)	14(36.8)	ref			
	Severe	43(67.2)	21(32.8)	1.19(0.52,2.77)	0.68		

## Discussion

This study aimed to determine retention in HIV care and associated factors among youths aged 15–24 years enrolled at Kabuyanda HC IV in Isingiro district, southwestern Uganda. We found that retention in HIV care was 65.7%. Youth who were male, married and had perinatally acquired HIV were significantly associated with retention in HIV care. HIV related stigma was not statistically significantly associated with retention in HIV care among our participants.

The level of retention in HIV care in our study is much lower than the UNAIDS target of 90%, but similar to that reported in other studies done in Uganda by Okoboi et al. [22], who found a rate of 65% among Ugandan adolescents aged 10–19 years. However, the retention in HIV care rate of 65.7% in our study is much higher than 29.3% that was reported by Izudi et al. [10] in central Uganda.

Our findings on retention in HIV care are lower than those reported by Nabukeera et al. [23] in their study to establish adherence to antiretroviral therapy and retention in HIV care for adolescents living with HIV from 10 districts in Uganda who reported a retention in HIV care rate of 90%. The difference may be due to differences in the characteristics of the study sample. While the study by Nabukeera et al. [23] recruited adolescents only (10–19 years), we recruited both adolescents (15–19 years) and young adults (20–24 years). The retention in HIV care rate in our study is also lower than that of Brown et al. [24] among youths 15–24 years in rural Kenya where 81% of these youths had been retained in HIV care at 1 year.

Youths who were married were significantly associated with retention in HIV care in our study. Our findings are similar to those reported by Umeokonkwo et al. [25] in Anambra state in Nigeria in which marital status was a significant predictor of retention in HIV care. Being married, or having a spouse provides a source of psychological and emotional support which improves retention in HIV care compared to those who are single and not in a relationship. In a study by Santos et al. [26] to determine the source of social support of people with HIV, the main source of physical, emotional and social support were spouses/partners. However, a study about predictors of retention in HIV care among youth (15–24 years) in a universal test-and-treat setting in rural Kenya, didn’t find any association between marital status and retention in HIV care [24].

Youth who perinatally acquired HIV are likely to be retained in HIV care. In a study in United Kingdom, among young adults with perinatally acquired HIV infection that assessed the clinical outcomes post transition to adult services, 86% of these youths were retained in HIV care [27]. In a systematic review by Ritchwood et al. [28] to examine retention in HIV care, it indicated that more than 70% of such adolescents who were infected with HIV perinatally were retained in care 1–2 years post-Health Care Transition. Such youths are better retained in care because of the special attention they receive during their paediatric HIV care, and the intense preparations for transitions into adult care [29]. These adolescents and young adults have been found to have a special patient-provider relationship that is constituted by a strong bond in between since the provider understands the patient in details [29]. There is an element of adaptation to the clinic environment, boosted by the individual experience [28].

In this study, males show a more likelihood of being retained in HIV care. This finding is similar to that reported by Valverde et al. [30], that showed that retention in care rates were lower for females than for males. This finding deviates from commonest findings from many studies that have been done, that instead show a female predilection of good retention in HIV care [31–33]. This improvement in male retention may be a result of the many interventions, modifications in care and recommendations of male involvement in elimination of mother to child transmission (eMTCT) services in Uganda [34, 35]. This integrated practice in Uganda reinforces, HIV treatment support, economic support and psychosocial support among the expectant couple [35]. Such services have been shown to improve male involvement, and hence indirectly impacting positively on their retention in HIV care.

Our study did not find an association between HIV related stigma and retention in HIV care. This is similar to what was found in other studies [36–38]. However, there are studies which showed that HIV related stigma was associated with retention in HIV care [39–45]. This may be because of differences in study designs as some of the studies were qualitative studies [44, 45]. These studies [39–42] were also carried out in a different setting compared to ours, with varying tools to measure HIV related stigma as well as varying age groups. This suggests that there may be geographic, cultural and age differences between these studies and our study. The difference in our findings could also be due to our strict definition of retention as some studies that found an association between HIV related stigma and retention in care have defined poor retention as going 12 months since the last documented clinic visit [43]. Some authors have also suggested that HIV related stigma may hinder initial linkage to care but play a lesser role on retention of patients in care [36]. This may explain the lack of association between HIV related stigma and retention in care for those already linked to care.

### Limitations

This study has some limitations. The Berger Stigma Scale used to assess stigma has commonly been used in the adult population. In Uganda, an adult is any person ≥18 years of age. However, in this study we had some participants aged below 18 years who were subjected to the same tool to assess stigma. The definition of retention that was used in this study was conservative and might potentially have underestimated the true retention of youths in care. In this study, we were not able to assess the association between retention and factors such as immunological/virologic status (CD4 count and HIV viral load measures) or ART duration, poverty/income. Perinatally acquired HIV was only assessed verbally. Finally, this study only employed a quantitative approach and therefore we did not collect information on patient’s reasons for not attending scheduled visits.

## Conclusion

Youths aged 15–24 years are still poorly retained in HIV care in rural southwestern Uganda despite the efforts put in place by the ministry of health to improve HIV care. Being male, having perinatally acquired HIV and married or in a relationship are associated with retention in HIV care.

### Recommendations

We recommend interventions specifically targeting adolescents and young adults living with HIV to improve retention in HIV care especially in rural settings. These interventions should focus on improving social and emotional support through the creation and enhancement of peer support projects and friendly services to optimize retention in HIV care for adolescents and young adults (15–24 years) who live with HIV in rural Uganda. There is need for further research studies to qualitatively identify the barriers and facilitators to retention of adolescents and young adults in HIV care services.

## Supplementary Information

**Additional file 1.**

## Data Availability

All the data needed for this manuscript has been included. In case there is a need for clarifications, the corresponding author can be contacted.

## Abbreviations

ART: Antiretroviral therapy
HC IV: Health Centre Four (IV)
HIV: Human Immunodeficiency Virus
UBOS: Uganda Bureau of Statistics
