# Supplementary material for: Retention in HIV care and associated factors among youths aged 15–24 years in rural southwestern Uganda
Source: BMC Public Health. 2021 Jul 31;21:1489. doi: 10.1186/s12889-021-11547-5 (PMC8325848; doi:10.1186/s12889-021-11547-5)
Supplement: Supplementary file 1 — Additional file 1. [file 12889_2021_11547_MOESM1_ESM.pdf]

QUESTIONNAIRE - ENGLISH VERSION

**Questions**	**Response**
How old are you (in complete years)?	
What is your biological sex?	Male
	Female
What is your current marital status?	Unmarried
	Married
What is your highest level of Education?	None
	Primary
	Secondary
	Tertiary
What is your subcounty of origin?	Kabuyanda SC
	Kabuyanda TC
	Kikagati SC
	Other
Have you ever disclosed your HIV status?	Yes

	No
Did you acquire HIV perinatally?	Yes
	No
Retention in HIV care : the total number of clinic visits in each quarter	1st quarter: ____________________
	2nd quarter: ___________________
	3rd quarter: ___________________
	4th quarter: ___________________
